# Bacterial Multiresistance and Microbial Diversity of Milk Received by a University Hospital Milk Bank

**DOI:** 10.3390/microorganisms13010028

**Published:** 2024-12-27

**Authors:** Dayane da Silva Zanini, Benedito Donizete Menozzi, Wanderson Sirley Reis Teixeira, Felipe Fornazari, Gismelli Cristiane Angeluci, Raquel Cuba Gaspar, Lucas Franco Miranda Ribeiro, Carlos Eduardo Fidelis, Marcos Veiga dos Santos, Juliano Gonçalves Pereira, Helio Langoni

**Affiliations:** 1 School of Veterinary Medicine and Animal Science, São Paulo State University (UNESP), Rua Prof. Doutor Walter Maurício Correa, s/n, Botucatu 18618-681, Brazil; d.zanini@unesp.br (D.d.S.Z.); juliano.pereira@unesp.br (J.G.P.); 2 Qualileite Laboratory, Department of Animal Nutrition and Production, University of São Paulo (USP), Avenida Duque de Caxias Norte, 225, Pirassununga 13635-900, Brazil

**Keywords:** breast milk, human milk bank, microbiota, multidrug resistance

## Abstract

Breastfeeding is fundamental for the development and protection of the newborn, and microorganisms present in breast milk are associated with the development of the infant’s intestinal microbiota. However, there are factors that interfere with breastfeeding, resulting in the need to supply donated milk to milk banks for these children. Even though there is a restriction on medications prescribed for pregnant and breastfeeding women, some antimicrobials are accepted, as long as they are used correctly and as they can increase the selection pressure for resistant bacteria. The microorganisms present in breast milk from a human milk bank were evaluated and the resistance of the isolates to antimicrobials was phenotypically characterized. In total, 184 microbial isolates were identified by mass spectrometry, of 12 bacterial genera and 1 yeast genus. There was a high prevalence of bacteria of the genus *Staphylococcus*, mainly *S. epidermidis* (33%). Resistance to antimicrobials varied among species, with a higher percentage of isolates resistant to penicillins and macrolides. Multidrug resistance was identified in 12.6% of 143 isolates. Breast milk contains a wide variety of microorganisms, mainly those of the *Staphylococcus* and *Enterobacter* genera. There was a high percentage of resistant isolates, and multidrug resistance in *Klebsiella oxytoca* (66.7%; 4/6) and *S. epidermidis* (15.0%; 9/60) isolates, which increases the public health concern.

## 1. Introduction

Public health agencies around the world, like the World Health Organization (WHO) and the United Nations Children’s Fund (UNICEF), recommend breastfeeding as the sole source of food, from the first minutes of life until six months old and its continuity until two years of age, with the addition of a varied diet [1].

Evidence shows that exclusive breastfeeding promotes benefits for children in addition to nutritional training, such as cognitive development and reduced risk of infectious and non-infectious diseases, with further benefits for the mother [2]. However, less than half the world’s children are exclusively breastfed, due to maternal social, physiological, or psychological factors or the infant’s health status [1].

If breastfeeding is impossible due to the mother’s health complications or if the newborn needs to be hospitalized and cannot be breastfed by the mother, human milk banks are an alternative for infant nutrition. The banks receive donations from lactating women who produce surplus milk. Before distribution to hospitalized children, the milk is assessed for its quality and is subsequently pasteurized [3].

Bacteria of the genera *Lactobacillus*, *Staphylococcus*, *Enterococcus*, and *Bifidobacterium* are part of the normal microbiota of human milk and are important for the development of the infant intestinal microbiota and for the protection of children against pathogens and other health complications [4]. However, the indiscriminate use of antibiotics, especially during breastfeeding, can cause damage that goes beyond the balance of this microbiota, since women who breastfeed and use antibiotics preventively to treat pre- and/or postpartum infections (cesarean sections), or even ingest products of animal origin with such residues [5,6], are a concern for global public health, mainly due to the growing bacterial resistance to antimicrobials [7].

The infant’s intestine is colonized by a variety of microorganisms that can undergo changes in quantity and diversity due to antimicrobial use, together with resistance genes carried by intestinal bacteria that certain findings suggest are transferred through breast milk [8], which occur in greater quantity at this stage and can increase with the use of medications [9,10].

Some species of the genus *Staphylococcus* are considered part of the microbiota of the skin and mucosa of humans and animals, while the main species of importance in public health is *S. aureus* [11] due to its ability to produce heat-resistant enterotoxins responsible for food poisoning [12].

Here, we detected and identified microorganisms from among human milk microbiota and investigated the profile of bacterial resistance to antimicrobials in samples received by a public milk bank.

## 2. Materials and Methods

### 2.1. Samples

The samples evaluated in this work came from a university hospital milk bank in the central region of the State of São Paulo and were sent to the Food Inspection Laboratory of the School of Veterinary Medicine and Animal Science, UNESP, Botucatu, São Paulo, from January to August 2023, for the Dornic acidity assay.

The analyzed milk was collected and stored according to the protocol established by the milk bank [13] and during all stages of the research there was no contact with the donors and no information was requested.

A total of 200 samples were selected and separated into aliquots of 2 mL to 3 mL of milk, which were reserved in microtubes and maintained at −20 °C until use.

### 2.2. Microbial Isolation

Each milk sample was plated, by streaking with 10 microliters of each sample without dilutions, on non-selective blood agar medium (Oxoid^®^, Thermo Fisher Scientific™, Hampshire, UK) plus 6% bovine blood, a nutrient medium that allows the growth of diverse microorganisms, and selective medium for Gram-negative bacteria, MacConkey agar (Oxoid^®^, Thermo Fisher Scientific™, Hampshire, UK), for the selection of enterobacteria, a group with species of clinical medical importance was then incubated in an oven under aerobic conditions, at 37 °C, for up to 72 h, with observation of microbial isolation every 24 h [14].

Plates that exhibited moderate to exuberant growth of pure colonies and different colony patterns on both culture media were selected. Each colony with different patterns was submitted to Gram stain for a previous separation between Gram-positive and Gram-negative according to the literature [15].

The isolates were stored in tubes containing nutrient broth and glycerol (5%) and they were kept at −80 °C.

### 2.3. Identification of the Microorganism Species by Mass Spectrometry

The microbial isolates stored at −80 °C were plated on blood agar and maintained at 37 °C, for 24 h. Next, the plates were sent to the milk quality research laboratory (Qualileite) of the School of Veterinary Medicine and Animal Science of the University of São Paulo for species identification using matrix-assisted laser desorption/ionization time-of-flight mass spectrometry -MALDI-TOF MS (Bruker Daltonics™, Bremen, Germany) [16,17].

From each plate, pure colonies were selected and treated with acidic solutions, according to the laboratory protocol [16,17].

Identification was performed by Autoflex III mass spectrometer (Bruker Daltonics™, Bremen, Germany) using FlexControl 3.3 software package and results were analyzed by Biotyper 3.0 software package (Bruker Daltonics™, Bremen, Germany).

### 2.4. In Vitro Sensitivity Profile to Antimicrobials

The samples were inoculated in sterile saline solution at optical turbidity on the McFarland scale (0.5). With the aid of a sterile swab, each suspension was plated on Müeller-Hinton agar (BD Difco™, Sparks, MD, USA) according to the agar diffusion method [18], and evaluations of drug sensitivity followed the rules of the Clinical and Laboratory Standards Institute (CLSI) [19].

CLSI defines sensitive (S) microorganisms as isolates predicted to be associated with a high chance of treatment success, resistant (R) as associated with a low chance of treatment success, and intermediate (I) microorganisms for which you need to increase the dose or concentration of the antimicrobial [20].

Nine antimicrobial agents from five different classes were evaluated: (1) penicillins and beta-lactam derivatives (amoxicillin 10 µg, ampicillin 10 µg, cephalexin 30 µg, ceftriaxone 30 µg), (2) macrolides (azithromycin 15 µg, erythromycin 15 µg), (3) lincosamides (clindamycin 2 µg), (4) aminoglycosides (gentamicin 10 µg), and (5) sulfonamides (sulfamethoxazole + trimethoprim 25 µg). Antibiotics that could be prescribed for pregnant and breastfeeding women were the sole criterion for selection [5].

Multidrug-resistant bacteria were considered when there was simultaneous resistance to three or more classes of the tested antimicrobials [6].

## 3. Results

### 3.1. Identification of Microorganisms

Microorganisms were isolated in 56.0% (112/200) of the samples selected. According to MALDI-TOF MS data, there were 13 identified microorganism genera branched into 30 species, 28 species of bacteria, and 2 of yeast, totaling 187 distinct microbial isolates (Table 1).

Regarding classification based on the bacterial cell wall, 3 Gram-positive genera were detected from the species *Staphylococcus* (*S. epidermidis*, *S. aureus*, *S. lugdunensis*), *Enterococcus faecalis*, and *Kocuria kristinae*, while in the Gram-negative class, 9 distinct genera and 23 species were detected (Table 1). Regarding prevalence, the most significant genera were *Acinetobacter* (5 spp.), *Pseudomonas* (5 spp.), *Enterobacter* (4 spp.), *Klebsiella* (3 spp.), and *Serratia* (3 spp.).

### 3.2. In Vitro Microbial Sensitivity Profile of Isolates

For the antimicrobials tested, the highest sensitivity was observed for gentamicin (77.9%; 134/172), followed by sulfamethoxazole + trimethoprim (62.8%; 108/172) and ceftriaxone (57.6%; 99/172). Regarding resistance, the most significant was azithromycin (45.4%; 78/172), followed by cephalexin (36.0%; 62/172) and amoxicillin (26.2%; 45/172). There were isolates that were intermediate (I) to the five classes of antimicrobials, ranging from 0.5% to 10% (Table 2).

The multidrug resistance (MDR) evaluation (Table 3) showed that 10.5% (18/172) of the total number of isolates tested by the agar diffusion method were resistant to three or more classes of the antimicrobials tested: *S. epidermidis*, 15.0% (9/60); *K. oxytoca*, 66.7% (4/6); *S. marcescens*, 25.0% (2/8); *K. pneumoniae*, 40.0% (2/5); and *S. aureus*, 14.3% (1/7).

A high rate of resistance to beta-lactam derivatives 69.4% (50/72) and macrolides 58.5% (31/53) was determined for Gram-negative species.

Some microbial isolates, obtained in this study, did not have a standard of reference for antimicrobial agents tested according to CLSI, and the determination of the antimicrobials was not obtained, as described in Table 3.

In general, the antibiotic that performed best for the microorganisms tested was gentamicin, with the highest percentage of resistance among *S. epidermidis* strains (21.0%). Multidrug resistance was observed in three bacterial genera (*Klebsiella* spp., *Serratia* sp., and *Staphylococcus* spp.), corresponding to 12.6% (18/143) of the total of species that have standard data from the CLSI [19] for more than three of the five classes of antimicrobials tested (Table 3).

## 4. Discussion

Mass spectrometry is a powerful technique for the positive identification of microorganisms, due to its sensitivity, selectivity, and ability to provide objective data. It enables comparisons with robust databases and differentiation among a wide variety of bacteria, some with the potential to cause harm to human health, as corroborated in this study with regard to the prevalence of the genus *Staphylococcus* (39.0%) and the species *S. epidermidis* (33.2%) among the isolates identified.

In other studies that identified the microbiota of breast milk using mass spectrometry, the presence of *S. epidermidis* has been frequently observed [21,22,23]. This colonization of *Staphylococcus* on the skin is known to influence the microbiota composition of breast milk. Through skin colonization, *Staphylococcus* are transported by carriers, transferring the microorganism to other environments, thus increasing the risk of infection by this bacteria. In addition to high virulence and a variety of infections, this group has the ability to develop resistance to antimicrobials, like the isolates described in this work, which presented multidrug resistance in 15.0% of *S. epidermidis* and 14.3% of *S. aureus*.

*Streptococcus*, *Lactobacillus*, *Bifidobacterium*, and other microorganisms have been previously isolated in human milk and in the intestinal flora of healthy individuals, highlighting the frequency of these genera in milk microbiota [10,24,25,26,27].

*Staphylococcus* and *Streptococcus* are the main genera described in the majority of studies on the microbiota of human milk, around 90%, followed by lactic acid bacteria and a broad range of Gram-negative microorganisms that includes numerous genera [28,29,30]. These microorganisms form part of the main phyla that compose the microbiota of human milk (Firmicutes and Proteobacteria), and their origin has been associated with the translocation of intestinal bacteria to the mammary glands via the lymphatic route and retrograde flow from the infant’s oral cavity and breast [31]. These may vary due to the influence of intrapartum antibiotics, obesity, and supplements to the mother’s diet [30,31,32].

The use of antimicrobials at this stage is restricted because some medications are not safe for the child, which limits treatment options for infections. In this study, we selected nine antibiotics that are widely prescribed for breastfeeding women and observed that up to 45.0% of the bacteria isolated showed resistance to azithromycin, while the penicillins presented lower efficacy against all bacterial genera, as reported by Salerno et al. [6].

Despite the variation in resistance of the *Staphylococcus* species isolated, *S. aureus* (28.0% to 85.0%), *S. epidermidis* (5.0% to 75.0%), and *S. lugdunensis* (25.0% to 75.0%), our highest resistance rates are similar to those reported by Chen et al. [33], Marín et al. [34], and Salerno et al. [6], in which 60.0% to 90.0% of staphylococci were resistant to penicillin and erythromycin.

The prevalence of the species *S. epidermidis* in the samples stands out because it presented microbial MDR in 15.0% of the isolates, even though the highest MDR percentage was for *Klebsiella* spp. (54.5%; 6/11). Begović et al. [35] observed MDR of *S. epidermidis* in 28.0% of their isolates, Marín et al. [32] in 64.4%, while in the study by Salerno et al. [6], MDR was present in 66.1% (123/186) of the total isolates.

Resistance to antimicrobials develops due to the presence of resistance genes in bacterial species, as reported by several authors [10,36,37,38]. The genes present in the infant intestinal microbiota resemble those detected in mothers’ milk, indicating the transfer of carrier bacteria through breastfeeding.

## 5. Conclusions

The microbiota of breast milk presented a wide variety of microorganisms. Despite the predominance of enterobacteria, which seems to indicate failure in obtaining and handling the milk, the detection of the genus *Staphylococcus* spp. is the most relevant from the viewpoint of public health. The identification of multiresistant bacteria indicates that it is important to review and possibly adjust the antimicrobial treatment protocol, in addition to educational activities for donor mothers regarding the process of obtaining milk.

## Figures and Tables

**Table 1:** Species and frequency of microorganisms identified by mass spectrometry (MALDI-TOF MS) in human milk samples from a university hospital milk bank.

Species	No. of Species Isolates/Total No. of Isolates (%)
*Staphylococcus epidermidis*	62/187 (33.16%)
*Staphylococcus aureus*	7/187 (3.74%)
*Staphylococcus lugdunensis*	4/187 (2.14%)
*Enterobacter xiangfangensis*	14/187 (7.49%)
*Enterobacter asburiae*	9/187 (4.81%)
*Enterobacter cloacae*	5/187 (2.67%)
*Enterobacter kobei*	1/187 (0.53%)
*Stenotrophomonas maltophilia*	13/187 (6.95%)
*Acinetobacter ursingii*	8/187 (4.28%)
*Acinetobacter* sp.	5/187 (2.67%)
*Acinetobacter junii*	2/187 (1.07%)
*Acinetobacter pittii*	2/187 (1.07%)
*Acinetobacter baumannii*	1/187 (0.53%)
*Acinetobacter johnsonii*	1/187 (0.53%)
*Serratia marcescens*	8/187 (4.28%)
*Serratia liquefaciens*	2/187 (1.07%)
*Serratia nematodiphila*	1/187 (0.53%)
*Klebsiella oxytoca*	6/187 (3.21%)
*Klebsiella pneumoniae*	5/187 (2.67%)
*Klebsiella variicola*	2/187 (1.07%)
*Candida parapsilosis*	5/187 (2.67%)
*Candida intermedia*	1/187 (0.53%)
*Pseudomonas aeruginosa*	5/187 (2.67%)
*Pseudomonas oryzihabitans*	3/187 (1.60%)
*Pseudomonas monteilii*	2/187 (1.07%)
*Pseudomonas putida*	2/187 (1.07%)
*Pseudomonas koreensis*	1/187 (0.53%)
*Pseudomonas* sp.	1/187 (0.53%)
*Pantoea* sp.	2/187 (1.07%)
*Chryseobacterium gambrini*	1/187 (0.53%)
*Enterococcus faecalis*	1/187 (0.53%)
*Kocuria kristinae*	1/187 (0.53%)
*Raoultella ornithinolytica*	1/187 (0.53%)
Not identified	3/187 (1.60%)
Total	187/187 (100%)

**Table 2:** In vitro microbial sensitivity profile of bacterial isolates detected in breast milk from a university hospital human milk bank.

Class	Antimicrobials	Sensitivity Profile/No. of Isolates (%)		
		S	I	R
Penicillins and beta-lactam derivatives	Amoxicillin	9/172 (5.23%)	-	45/172 (26.16%)
	Ampicillin	1/172 (0.58%)	-	3/172 (1.74%)
	Cephalexin	62/172 (36.04%)	-	62/172 (36.04%)
	Ceftriaxone	99/172 (57.56%)	18/172 (10.47%)	26/172 (15.12%)
Macrolides	Azithromycin	45/172 (26.16%)	1/172 (0.58%)	78/172 (45.35%)
	Erythromycin	15/172 (8.72%)	2/172 (1.16%)	55/172 (31.98%)
Lincosamides	Clindamycin	63/172 (36.63%)	5/172 (2.91%)	3/172 (1.74%)
Aminoglycosides	Gentamicin	134/172 (77.91%)	8/172 (4.65%)	15/172 (8.72%)
Sulfonamides	Sulfamethoxazole + trimethoprim	108/172 (62.79%)	11/172 (6.40%)	37/172 (21.51%)

S: Sensitive, I: Intermediate, R: Resistant.

**Table 3:** In vitro resistance profile to antimicrobials of eight bacterial genera isolated from breast milk from a university hospital human milk bank.

Species	Resistance Profile (Resistance Frequency/no. and %)									
	Class/Antimicrobials									
	Penicillins and Beta-Lactam Derivatives				Macrolides		Lincosamides	Aminoglycosides	Sulfonamides	
	AMO	AMP	CEP	CEF	AZI	ERY	CLI	GEN	SUT	MDR
*Acinetobacter* spp.	0	0	0	2/19 (10.5%)	0	0	0	0/19 (0%)	4/19 (21.1%)	-
*Enterobacter asburiae*	8/9 (88.9%)	0	8/9 (88.9%)	2/9 (22.2%)	4/9 (44.4%)	0	0	1/9 (11.1%)	3/9 (33.3%)	-
*Enterobacter cloacae*	4/5 (80%)	0	4/5 (80.0%)	0/5 (0%)	1/5 (20.0%)	0	0	0/5 (0%)	0/5 (0%)	-
*Enterobacter xiangfangensis*	14/14 (100%)	0	13/14 (92.9%)	3/14 (21.4%)	8/14 (57.1%)	0	0	0/14 (0%)	6/14 (42.9%)	-
*Enterococcus faecalis*	0/1 (0%)	0/1 (0%)	0	0	0	0/1 (0%)	0	0	0	-
*Klebsiella oxytoca*	5/6 (83.3%)	0	2/6 (33.3%)	2/6 (33.3%)	4/6 (66.7%)	0	0	0/6 (0%)	4/6 (66.7%)	4/6 (66.7%)
*Klebsiella pneumoniae*	5/5 (100%)	0	1/5 (20.0%)	1/5 (20.0%)	5/5 (100%)	0	0	0/5 (0%)	2/5 (40.0%)	2/5 (40.0%)
*Klebsiella variicola*	1/2 (50.0%)	0	0/2 (0%)	0/2 (0%)	1/2 (50.0%)	0/2 (0%)	0	0/2 (0%)	1/2 (50.0%)	-
*Pseudomonas* spp.	0	0	0	0	0	0	0	0/14 (0%)	0	-
*Raoultella ornithinolytica*	1/1 (100%)	0	0/1 (0%)	0/1 (0%)	0/1 (0%)	0	0	0/1 (0%)	1/1 (100%)	-
*Serratia liquefaciens*	0/2 (0%)	2/2 (100%)	2/2 (100%)	0/2 (0%)	0/2 (0%)	0	0	0/2 (0%)	0/2 (0%)	-
*Serratia marcescens*	6/8 (75.0%)	0	7/8 (87.5%)	3/8 (37.5%)	7/8 (87.5%)	0	0	1/8 (12.5%)	2/8 (25.0%)	2/8 (25.0%)
*Serratia nematodiphila*	1/1 (100%)	1/1 (100%)	1/1 (100%)	0/1 (0%)	1/1 (100%)	0	0	0/1 (0%)	0/1 (0%)	-
*Staphylococcus aureus*	0	0	4/7 (57.1%)	2/7 (28.6%)	3/7 (42.9%)	6/7 (85.7%)	0/7 (0%)	0/7 (0%)	1/7 (14.3%)	1/7 (14.3%)
*Staphylococcus epidermidis*	0	0	19/60 (31.7%)	11/60 (18.3%)	42/60 (70%)	45/60 (75%)	3/60 (5.0%)	13/60 (21.7%)	7/60 (11.7%)	9/60 (15.0%)
*Staphylococcus lugdunensis*	0	0	1/4 (25.0%)	0/4 (0%)	2/4 (50.0%)	3/4 (75.0%)	0/4 (0%)	0/4 (0%)	0/4 (0%)	-
*Stenotrophomonas maltophilia*	0	0	0	0	0	0	0	0	6/13 (46.2%)	-

MDR: multidrug resistant. AMO: Amoxicillin (10 µg); AMP: Ampicillin (10 µg); CEP: Cephalexin (30 µg); CEF: Ceftriaxone (30 µg); AZI: Azithromycin (15 µg); ERY: Erythromycin (15 µg); CLI: Clindamycin (2 µg); GEN: Gentamicin (10 µg); SUT: Sulfamethoxazole + trimethoprim (25 µg). *Acinetobacter* spp.: *Acinetobacter* spp. (no = 5), *A. baumannii* (no = 1), *A. johnsonii* (no = 1), *A. junii* (no = 2), *A. pittii* (no = 2), *A. ursingii* (no = 8); *Pseudomonas* spp.: *Pseudomonas* spp. (no = 1), *P. putida* (no = 2), *P. oryzihabitans* (no = 3), *P. monteilii* (no = 2), *P. koreensis* (no = 1), *P. aeruginosa* (no = 5).

## Data Availability

All raw data relating to the report are available in the manuscript (Table 1, Table 2 and Table 3).
